# Metabolic characteristics of transmembrane prolyl 4-hydroxylase (P4H-TM) deficient mice

**DOI:** 10.1007/s00424-024-02920-5

**Published:** 2024-02-24

**Authors:** Tuulia Ala-Nisula, Riikka Halmetoja, Henri Leinonen, Margareta Kurkela, Henna-Riikka Lipponen, Samuli Sakko, Mikko Karpale, Antti M. Salo, Niina Sissala, Tapio Röning, Ghulam S. Raza, Kari A. Mäkelä, Jérôme Thevenot, Karl-Heinz Herzig, Raisa Serpi, Johanna Myllyharju, Heikki Tanila, Peppi Koivunen, Elitsa Y. Dimova

**Affiliations:** 1 Biocenter Oulu, Faculty of Biochemistry and Molecular Medicine, Oulu Center for Cell-Matrix Research, University of Oulu, Aapistie 7C, P.O. Box 5400, 90014 Oulu, Finland; 2 A.I. Virtanen Institute for Molecular Sciences, University of Eastern Finland, Kuopio, Finland; 3 School of Pharmacy, University of Eastern Finland, Kuopio, Finland; 4 Research Unit of Biomedicine and Internal Medicine, Biocenter Oulu, Medical Research Center and University Hospital, Oulu, Finland; 5 Research Unit of Health Sciences and Technology, University of Oulu, Oulu, Finland

**Keywords:** Transmembrane prolyl 4-hydroxylase, Glucose metabolism, HIDEA syndrome

## Abstract

Transmembrane prolyl 4-hydroxylase (P4H-TM) is an enigmatic enzyme whose cellular function and primary substrate remain to be identified. Its loss-of-function mutations cause a severe neurological HIDEA syndrome with hypotonia, intellectual disability, dysautonomia and hypoventilation. Previously, P4H-TM deficiency in mice was associated with reduced atherogenesis and lower serum triglyceride levels. Here, we characterized the glucose and lipid metabolism of *P4h-tm*^*−/−*^ mice in physiological and tissue analyses. *P4h-tm*^*−/−*^ mice showed variations in 24-h oscillations of energy expenditure, VO_2_ and VCO_2_ and locomotor activity compared to wild-type (WT) mice. Their rearing activity was reduced, and they showed significant muscle weakness and compromised coordination. Sedated *P4h-tm*^*−/−*^ mice had better glucose tolerance, lower fasting insulin levels, higher fasting lactate levels and lower fasting free fatty acid levels compared to WT. These alterations were not present in conscious *P4h-tm*^*−/−*^ mice. Fasted *P4h-tm*^*−/−*^ mice presented with faster hepatic glycogenolysis. The respiratory rate of conscious *P4h-tm*^*−/−*^ mice was significantly lower compared to the WT, the decrease being further exacerbated by sedation and associated with acidosis and a reduced ventilatory response to both hypoxia and hypercapnia. P4H-TM deficiency in mice is associated with alterations in whole-body energy metabolism, day-night rhythm of activity, glucose homeostasis and neuromuscular and respiratory functions. Although the underlying mechanism(s) are not yet fully understood, the phenotype appears to have neurological origins, controlled by brain and central nervous system circuits. The phenotype of *P4h-tm*^*−/−*^ mice recapitulates some of the symptoms of HIDEA patients, making this mouse model a valuable tool to study and develop tailored therapies.

**Supplementary Information:**

The online version contains supplementary material available at 10.1007/s00424-024-02920-5.

## Introduction

The highly conserved hypoxia-inducible factor (HIF) signaling pathway couples oxygen sensing with transcriptional control. Hypoxia-inducible factor prolyl 4-hydroxylase 1 (HIF-P4H-1, alternatively named as, EglN2, PHD1), hypoxia-inducible factor prolyl 4-hydroxylases 2 (HIF-P4H-2, also known as EglN1, PHD2) and hypoxia-inducible factor prolyl 4-hydroxylases 3 (HIF-P4H-3, also referred to as EglN3 and PHD3) are enzymes that act as cellular oxygen sensors and belong to the 2-oxoglutarate (2OG)-dependent oxygenase superfamily [8, 10]. HIF-P4Hs 1–3 catalyze the hydroxylation of proline residues of the HIFα subunit using molecular oxygen and 2OG as co-substrates and Fe(II) as a cofactor [27]. This directs HIF to proteasomal degradation [10]. When oxygen levels decline, HIF-P4Hs’ activity decreases, leading to stabilization of HIFα, and formation of an active transcription dimer with the β-subunit that regulates > 300 genes to balance the oxygen supply and demand [10, 13, 28]. One of the key processes regulated by HIF is the reprogramming of energy metabolism including up-regulation of the oxygen-independent glycolysis and down-regulation of the O_2_-demanding mitochondrial oxidative phosphorylation [13].

Transmembrane prolyl 4-hydroxylase (P4H-TM), considered to be the fourth HIF-P4H [14, 23], is a unique prolyl 4-hydroxylase whose primary substrate and main cellular function are still unknown. P4H-TM is located at the endoplasmic reticulum (ER) membrane with the catalytic domain in the ER lumen [14]. It has a unique EF domain and its catalytic activity may be regulated by Ca^2+^ [22]. Although P4H-TM has been shown to hydroxylate HIFα in vitro [14, 23] and regulate HIFα protein levels oxygen-dependently in cortical neurons [17] and calcium signaling via HIF1-dependent mechanism in murine astrocytes [4], functional and structural data suggest that HIFα is not its primary substrate [14, 17, 18, 22]. In humans, loss-of-function mutations in *P4HTM* are associated with HIDEA syndrome, a neurological phenotype characterized by muscle and central hypotonia, hypoventilation, sleep apnea, intellectual disability, dysautonomia, epilepsy and eye abnormalities [9, 15, 24], and more recently with syndromic obesity [26].

In a previous study, we showed that *P4h-tm*^*−/−*^ mice on a *Ldlr*^*−/−*^ background are less prone to atherosclerosis development due to immuno-metabolic mechanisms associated with P4H-TM deficiency and partly attributed to their lower serum triglyceride (TG) levels on both a standard laboratory diet and a high-fat diet [21]. Low TG levels can lead to altered energy metabolism. The reported effects of the genetic deficiency of the three classical HIF-P4Hs on metabolism are significant ([29] and references therein), however, knowledge of the involvement of P4H-TM is limited. Therefore, we set out to investigate the role of P4H-TM in glucose and lipid metabolism.

## Experimental procedures

### Animal experiments

The *P4h-tm*^*−/−*^ mice were generated as described [16, 17]. All mice were fed normal chow (6.2% fat, 44.2% carbohydrate, 18.6% protein [w/w], Harlan Teklad, USA) ad libitum and were maintained on a normal 12-h light/dark cycle. Both sexes were used in the experiments.

### Metabolic profiling

Energy expenditure, locomotor and rearing activities, O_2_ consumption and CO_2_ production, food and water intake were monitored using an automated home cage phenotyping system (PhenoMaster, TSE Systems GmbH, Germany). The mice were individually housed in a controlled environment of 12 h light:dark (lights on 06:00–18:00) and temperature (21.5–22.5 °C) and acclimated to the home cage for a week prior to data collection. Mice were then monitored in the home cage system for a week provided with food and water ad libitum. Locomotor activity was measured using a multidimensional infrared light-beam detection system. Continuous food and water intake was recorded using lid-mounted sensors.

### Glucose and insulin tolerance tests and determination of serum insulin levels

Glucose tolerance test (GTT) and insulin tolerance test (ITT) were performed on both male and female mice after a 12 h overnight fast with or without sedation with fentanyl/midazolam (0.1 ml/10 g) (Fentanyl 50 µg/ml, Midazolam 5 mg/ml). For the GTT under sedation, mice were injected intraperitoneally with 1 g/kg glucose, and blood glucose concentrations were monitored from the *vena saphena* using a glucometer (Contour, Bayer). In the GTT without anesthesia, blood samples were taken from a mouse tail by tail-snip method [2]. Serum insulin levels were determined using the Ultra Sensitive Mouse Insulin ELISA Kit (90080, Crystal Chem), and HOMA-IR scores were calculated from the glucose and insulin values. For the ITT, mice were injected intraperitoneally with 1 IU/kg insulin (Humulin Regular, Lilly), and the blood glucose concentrations were determined as for the GTT.

### Glucagon tolerance test and determination of serum glucagon levels

Glucagon tolerance test was performed on 3–4-month-old male and female mice after a 6 h fast without sedation. The mice were intraperitoneally injected with 20 µg/kg glucagon (Glucagen©, Novo Nordisk A/S) and blood glucose concentrations were monitored from mouse tail by tail-snip method [2] at 15, 30, 60 and 120 min time points. Serum glucagon levels were determined using a glucagon ELISA kit (81518, CrystalChem) according to the manufacturer’s instructions.

### Determination of blood lactate, serum free fatty acids and free glycerol levels

The blood lactate concentrations were determined with a lactometer (Lactate Scout + , SensLab/EKF Diagnostics). Serum free fatty acids (FFAs) were measured using a fluorometric FFA assay kit (ab65341, Abcam), according to the manufacturer’s instructions, without freezing.

### Determination of tissue glycogen

100 mg of liver’s superior lobe or skeletal muscle *(M. quadriceps femoris)* from fed or 12 h fasted mice were homogenized, and the supernatant was assayed with a fluorometric Glycogen Assay Kit according to the manufacturer’s instructions (700480, Cayman Chemical).

### Determination of PEPCK activity

Liver’s superior lobe (50 mg) or a kidney of 12 h overnight fasted mice terminally anesthetized with fentanyl/fluanisone and midazolam were homogenized and the supernatant assayed with a colorimetric Phosphoenolpyruvate Carboxykinase Activity Assay Kit according to the manufacturer’s instructions (MAK408, Sigma-Aldrich).

### Respiratory response to hypoxia, hypercapnia and sedation

The respiratory response to hypoxia and hypercapnia was performed on conscious 5-month-old male mice. The mice were trained to a 1L glass chamber and the experimental set-up five times during the previous week. Mice were allowed to adjust for 15 min before the baseline respiratory rate was recorded for 2 min. The chamber was then flushed with a hypoxic (10% O_2_) or hypercapnic (5% CO_2_) gas mixture. The desired gas concentrations were achieved in ~ 8 min. O_2_ and CO_2_ levels were continuously monitored via O_2_ and CO_2_ sensors. The mice were kept in 10% O_2_ or 5% CO_2_ for 10 min, the last 2 min of which were video recorded. Between each mouse the chamber was left open for 5–10 min to ventilate back to normoxia (21% O_2_, 79% N_2_ and < 0.3% CO_2_). The mice had a 2-week recovery period between hypoxia and hypercapnia experiment. In another set of experiments after recording the baseline respiratory rate, the 3-month-old mice were sedated intraperitoneally with fentanyl/midazolam (0.1 ml/10 g) (Fentanyl 50 µg/ml, Midazolam 5 mg/ml) and their respiratory rate was recorded for a 2 min period after the onset of sedation. The respiratory rate was counted from the videos from five short time lapses that were slowed down and the average rate was used for the results.

### Grid-hanging test

This test was used to determine static muscle force. The mouse was placed on a 20 cm × 25 cm wire grid (grid unit 1 cm by 1 cm) that was carefully placed upside down as the lid of a 24 cm × 35.5 cm × 24 cm transparent plastic cage. The time to fall off until a cut-off time of 300 s was measured with a stop-watch. The test was repeated three times with a 10-min interval between the trials and the mean of all trials was recorded. After the test, the mice were returned to a group cage.

### Rotarod

The test measures dynamic muscle force, motor coordination, and balance. About 45 min before the actual experiment, the mice were allowed to practice balancing on the rod (Mouse Rota-Rod 47,600, Ugo Basile, Italy) for 2 min at 8 rpm rotation speed. If the mouse fell off the rod, it was lifted back on it. Two mice were tested simultaneously. The experiment itself consisted of three rounds with 30 min of resting time between each round. First, the mouse was placed on a stationary rod for 30 s. Then the rod started to rotate slowly at 5 rpm for 30 s. Thereafter, the speed was increased during the next 6 min from 5 to 24 rpm. Latency to fall off the rod was recorded. The cut-off time for the test was set at 6 min. If the mouse spun on the rod without taking any steps for three rounds, its test was terminated. The mean of three consecutive rounds was calculated.

### GatWalk gait analyses

Possible gait alternations were measured by CatWalk XT 9.1 (Noldus, Wageningen, the Netherlands), an automated gait analysis system. Mice were let run freely through a 130 cm long alley with a glass plate floor. A high-speed camera recorded the paw contacts from below, and the program analyzed numerous static and dynamic parameters assessing individual paw functioning and gait patterns. We recorded three uninterrupted runs through the alley and selected the following parameters for analysis: print area, stride length, and swing speed for each paw, and base of support for forepaws and hindpaws. These parameters were initially calculated for each run and for each paw, then averaged over the runs.

### Statistical analyses

Student’s two-tailed t test was used for the statistical significance of differences between two groups. Mann–Whitney U-test was used for the grid-hanging test. GraphPad Prism version 10.0.0 for Windows, GraphPad Software, Boston, Massachusetts USA, http://www.graphpad.com was used to analyze the data and generate the figures. All data are presented as mean ± standard error of the mean (SEM). *p* < 0.05 was considered statistically significant.

## Results

### Anthropometric analyses of *P4h-tm*^*−/−*^ mice

To evaluate the impact of P4H-TM deficiency on whole body metabolism, we followed body weight of the mice fed normal chow once a month until 1 year of age. While the weight gain was normal in both genotypes, we observed a significant ~ 10% reduction in body weight of the *P4h-tm*^*−/−*^ male mice until 8 months compared to WT (Fig. S1a). No differences in body weight were observed between the genotypes in female mice (Fig. S1b).

At sacrifice, there were no significant differences in the weights of gonadal WAT, liver, spleen or brown adipose tissue (BAT) between the genotypes of 1-year-old male mice (Fig. S1a). However, compared to WT mice, there was a ~ 10% increase in the average weight of the kidneys in *P4h-tm*^*−/−*^ males (*P* = 0.014, Fig. S1a).

At sacrifice, no differences in the weights of gonadal WAT, liver or kidney were observed between the genotypes in 1-year-old female mice, whilst the female *P4h-tm*^*−/−*^ mice were found to have heavier spleen and BAT than WT by ~ 20% (*P* = 0.047) and 40% (*P* = 0.034), respectively (Fig. S1b).

These results indicate that deficiency of P4H-TM may have some sex-specific effects on body and organ weights, which will be studied further.

### Metabolic profile of *P4h-tm*^*−/−*^ mice shows subtle disturbances in 24-h oscillations of energy expenditure, locomotor and rearing activities, O_2_ consumption and CO_2_ production

**Fig. 1:**
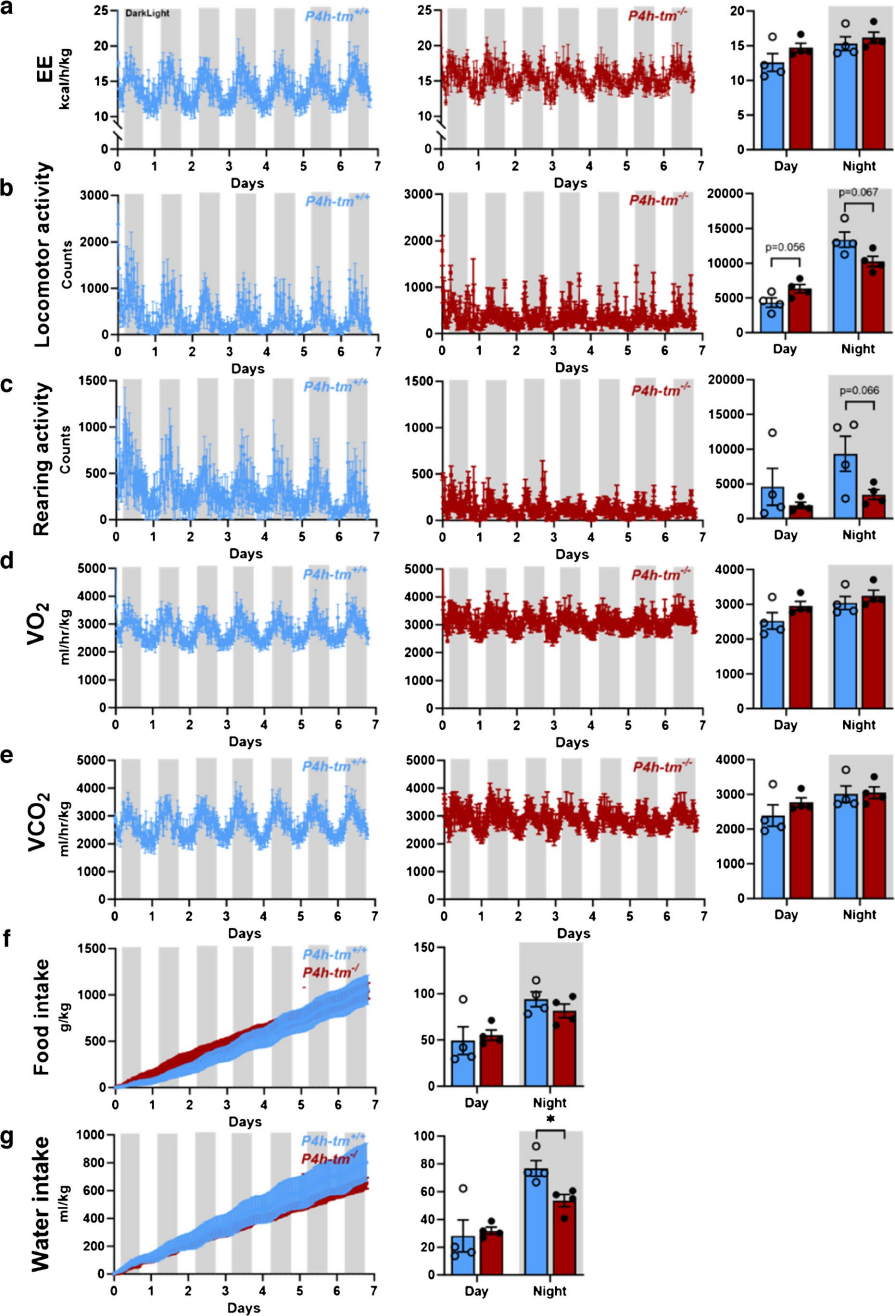
*P4h-tm*^*−/−*^ mice show subtle variations in frequency and a reduced amplitude in diurnal oscillations of energy expenditure, locomotor and rearing activity, O_2_ consumption and CO_2_ production and appear to be more active during the day. **(a-g)** Metabolic profiling of 5-month-old males housed in a home cage phenotyping system for 7 days at room temperature and a normal light/dark rhythm. (**a**) Energy expenditure (EE), (**b**) Locomotor activity, (**c**) Rearing activity, (**d**) O_2_ consumption (VO_2_), (**e**) CO_2_ production (VCO_2_), (**f**) Food intake, (**g**) Water intake. Data are mean ± SEM. *p ≤ 0.05, *n* = 4 *P4h-tm*^+*/*+^, *n* = 4 *P4h-tm*^*−/−*^

Next, we examined the effects of P4H-TM deficiency on whole-body energy homeostasis in mice using home cage analyses. The effect of P4H-TM deficiency on the metabolic rate was evaluated by calculating the energy expenditure (EE) from the data obtained by indirect calorimetry (Fig. 1a and Fig. S2a). Analyses of EE showed that there were no significant genotype differences in the male mice during both light and dark cycles. However, *P4h-tm*^*−/−*^ females had a higher EE during the day than the WT mice (Fig. S2a). Interestingly, the 24-h oscillations in EE of both male and female *P4h-tm*^*−/ −*^ mice were increased in frequency and reduced in amplitude compared to the WT littermates (Fig. 1a and Fig. S2a).

**Fig. 2:**
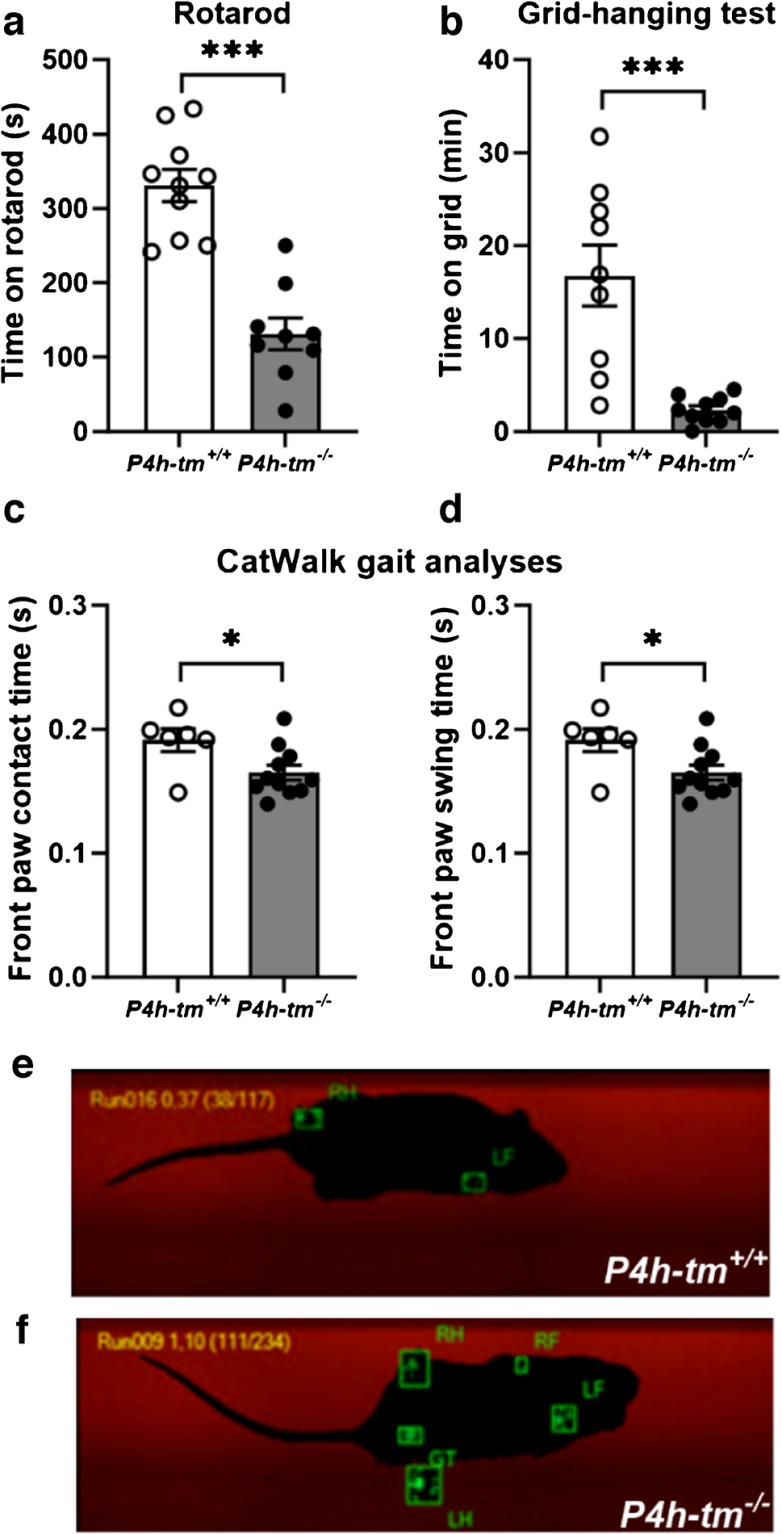
*P4h-tm*^*−/−*^ mice have muscle weakness and compromised motor coordination. (**a**) Grid-hanging test. (**b)** Rotarod test. (**c)** CatWalk gait analysis. (**e–f**) Snapshots of representative CatWalk runs of a *P4h-tm*^*−/−*^ (**e**) and a WT mouse (**f**). RH, right hind limb; RF, right front limb; LF, left front limb; LH, left hind limb; GT, genitals. Mann–Whitney U-test (Grid test): ***p < 0.001. T-test (Rotarod and Catwalk): *p ≤ 0.05, ***p < 0.001. *n* = 10 *P4h-tm*^+*/*+^, *n* = 10 *P4h-tm*^*−/−*^

In WT mice, locomotor activity during the day and at night followed repeated active and resting phases with a rapid increase at the onset of the dark phase (Fig. 1b and Fig. S2b). However, the *P4h-tm*^*−/−*^ mice appeared to be more active during the day than WT mice (*P* = 0.056 for males, *P* = 0.021 for females), and possibly less active than WT mice during the night (*P* = 0.067 for males) (Fig. 1b and Fig. S2b). Interestingly, the rearing activity, *e.g.* the act of standing on hind legs with or without support, was markedly reduced in both male and female *P4h-tm*^*−/−*^ mice (Fig. 1c and Fig. S2c). To assess the effect of P4H-TM deficiency on respiratory parameters, we recorded the oxygen consumption (VO_2_) and carbon dioxide production (VCO_2_) during the indirect calorimetry. A day-night rhythm in VO_2_ and VCO_2_ was observed in all mice. Again, however, the 24-h oscillations in VO_2_ and VCO_2_ of both male and female *P4h-tm*^*−/−*^ mice were increased in frequency and reduced in amplitude compared to their WT controls (Fig. 1d,e and Fig. S2d,e). Although the total food and water intake did not differ between the male genotypes, the *P4h-tm*^*−/−*^ males had a decreased water intake at night (*P* = 0.017, Fig. 1f,g). *P4h-tm*^*−/−*^ females did not differ from WTs in total food intake but had a lower water intake (Fig. S2f,g). These variations in food and drink intake could be due, at least in part, to reduced rearing activity and difficulty in reaching the food and drink bottle. Moreover, they also had decreased food (*P* = 0.02) and water intake (*P* = 0.003) during the night (Fig. S2f,g). These data suggest that *P4h-tm*^*−/−*^ mice, independent of sex, show variations in 24-h oscillations in EE, locomotor and rearing activity and VO_2_ and VCO_2_ compared to WT that may mimic dysautonomia and hypotonia of the HIDEA patients [9]. To further test this, we assessed neuromuscular function using a rotarod and grid-hanging test, and CatWalk gait analyses. *P4h-tm*^*−/−*^ mice could only maintain balance on an accelerating rotating rod for half of the time of the WT (*P* = 0.000003, Fig. 2a). Additionally, *P4h-tm*^*−/−*^ mice fell off from the upside-down grid during the first 0–3 min whereas the WT mice could hold the grip for several minutes (*P* = 0.0003, Fig. 2b). CatWalk gait analysis showed that *P4h-tm*^*−/−*^ mice had a shorter front paw contact time (*P* = 0.027, Fig. 2c,f) and swing time than WT mice (*P* = 0.025, Fig. 2d,e), which points to a rear-weighted posture, probably due to weakness of the leg muscles. In addition, *P4h-tm*^*−/−*^ mice were unable to run on a treadmill (Suppl. Video 1, 2). Altogether, these data suggest that the *P4h-tm*^*−/−*^ mice have both static and dynamic muscle weakness and impaired coordination resembling the HIDEA patients.

### Glucose tolerance and insulin sensitivity are increased in sedated *P4h-tm*^*−/−*^ mice

**Fig. 3:**
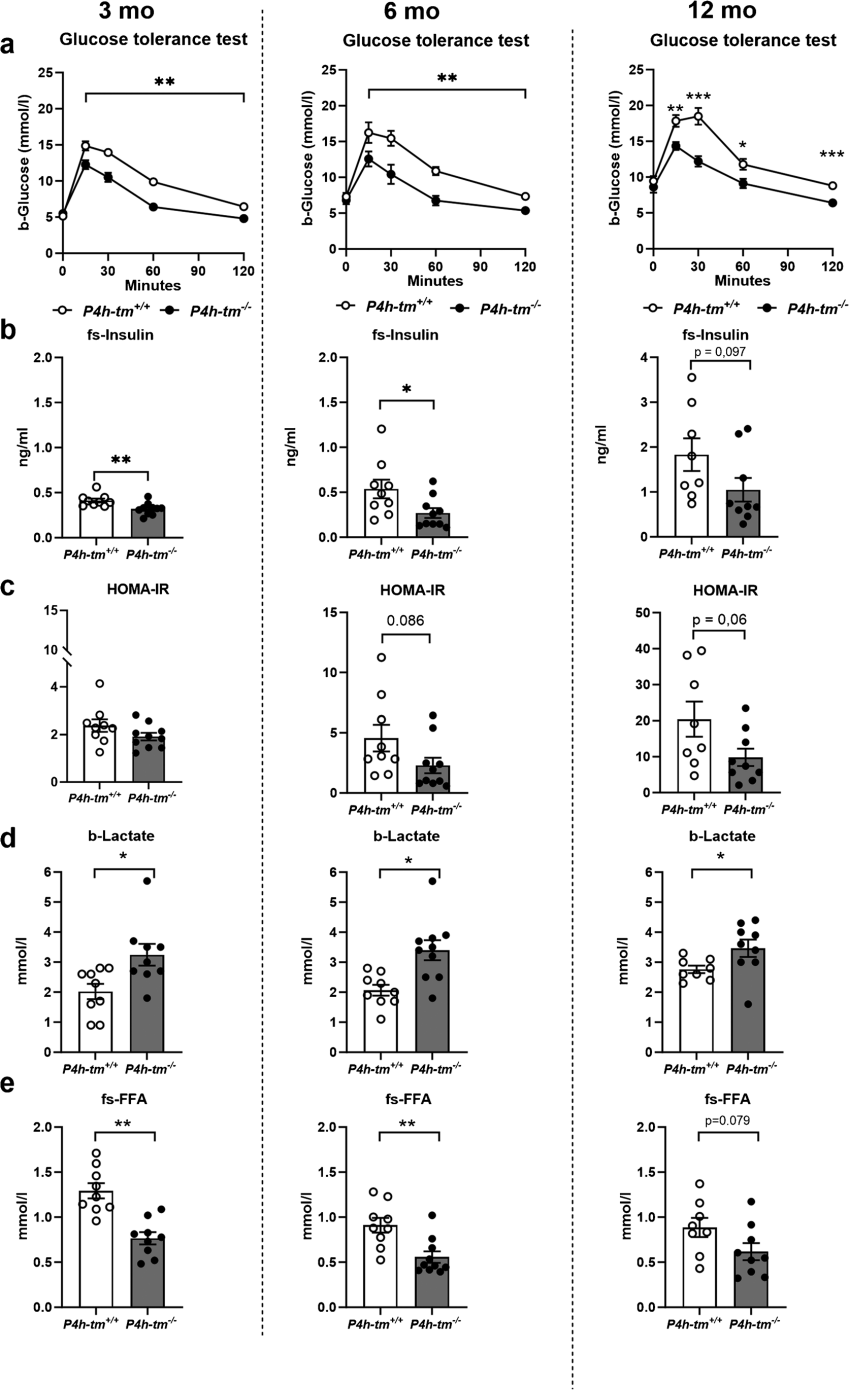
Sedated *P4h-tm*^*−/−*^ have improved glucose tolerance, lower insulin levels and HOMA-IR scores, increased blood lactate levels and decreased serum FFA levels. **(a)** Glucose tolerance test of 3-, 6- and 12-month-old male *P4h-tm*^+*/*+^and *P4h-tm*^*−/−*^ mice. The 0 min value was determined after 12 h fasting and sedation with fentanyl-midazolam. (**b**) Serum insulin levels, and (**c**) HOMA-IR values determined from the 0 min samples. (**d**) Fasting blood lactate levels. (**e**) Fasting serum FFA levels. Data are mean ± SEM. *p ≤ 0.05, **p < 0.01, ***p < 0.001. 3 mo *n* = 9 *P4h-tm*^+*/*+^, *n* = 10 *P4h-tm*^*−/−*^; 6 mo *n* = 9 *P4h-tm*^+*/*+^*,* *n* = 10 *P4h-tm*^+*/*+^; 12 mo *n* = 8 *P4h-tm*^+*/*+^*,* *n* = 9 *P4h-tm*^*−/−*^*.* b, blood, fs, fasting serum

To determine the physiological consequences of P4H-TM deficiency on glucose metabolism, we carried out intraperitoneal GTT and ITT on *P4h-tm*^*−/−*^ mice and their WT littermates. As glucose intolerance is associated with aging [11], we studied the glucose tolerance in a cohort of male mice at the age of 3, 6 and 12 months (Fig. 3). No differences in overnight fasting glucose levels were found between the genotypes in any of the age groups studied under fentanyl-midazolam sedation (Fig. 3a). However, *P4h-tm*^*−/−*^ mice had a better glucose tolerance, as evidenced by the faster clearance of glucose at all ages (Fig. 3a). The *P4h-tm*^*−/−*^ mice had significantly lower fasting serum insulin levels by ~ 25% at 3 months (*P* = 0.007), 50% at 6 months (*P* = 0.03) and 40% at 12 months (*P* = 0.097) than their WT littermates (Fig. 3b). Although the HOMA-IR scores increased by aging in both genotypes, they were ~ 50% lower in the *P4h-tm*^*−/−*^ mice at both 6 (*P* = 0.086) and 12 months (*P* = 0.06) in comparison to WT, yet this did not reach statistical significance (Fig. 3c). In addition, compared to WT, *P4h-tm*^*−/−*^ mice had significantly higher fasting blood lactate levels (50% higher at 3 months (*P* = 0.03), ~ 65% higher at 6 months (*P* = 0.03) and 25% higher at 12 months (*P* = 0.049) (Fig. 3d), and significantly lower fasting serum FFA levels (~ 40% lower at 3 (*P* = 0.002) and 6 months (*P* = 0.003) and 30% lower at 12 months (*P* = 0.079) (Fig. 3e).

Although female mice are often excluded from diabetes research being less glucose intolerant and less insulin-resistant than males [12], we studied a 1-year-old female cohort. As in males, we found similar trends toward difference in glucose tolerance (Fig. S3a), fasting serum insulin levels (Fig. S3b) and fasting blood lactate levels between the *P4h-tm*^*−/−*^ and WT females (Fig. S3d). No difference between the genotypes was found in HOMA-IR (Fig. S3c) while the *P4h-tm*^*−/−*^ females had significantly lower fasting serum FFA levels compared to WT (*P* = 0.0003, Fig. S3e).

**Fig. 4:**
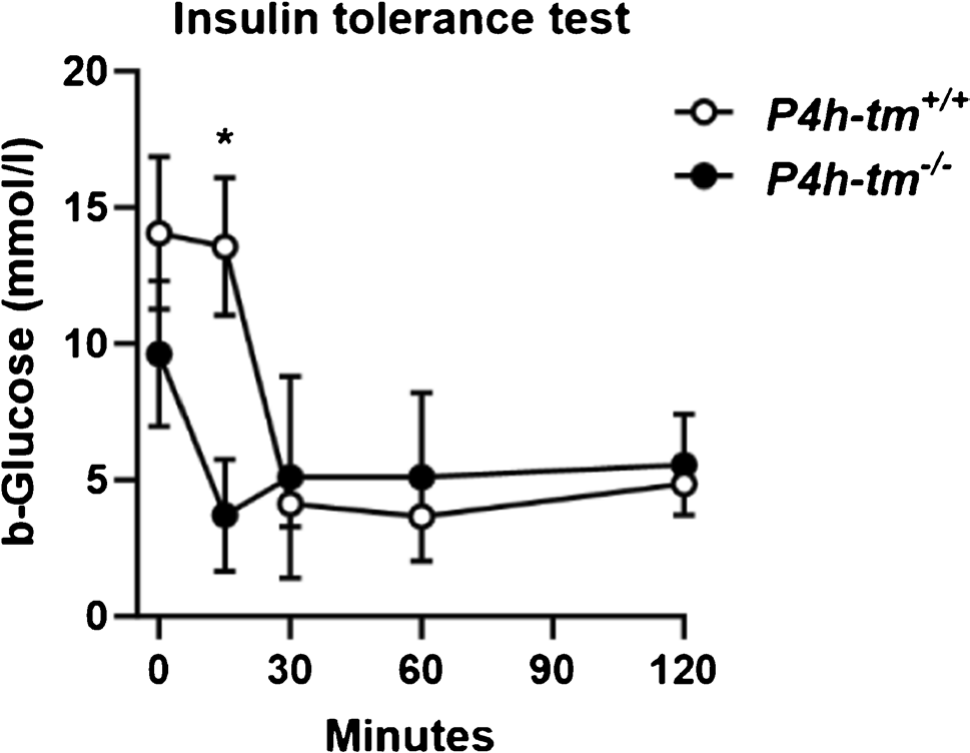
Sedated *P4h-tm*^*−/−*^ mice have improved insulin sensitivity. Insulin tolerance test of 6-month-old male *P4h-tm*^+*/*+^and *P4h-tm*^*−/−*^ mice. The 0 min value was determined after 6 h fasting and sedation with fentanyl-midazolam. Data are mean ± SEM. *p ≤ 0.05. *n* = 4 *P4h-tm*^+*/*+^*,* *n* = 4 *P4h-tm*^*−/−*^

Next, the whole-body insulin sensitivity of the mice was tested in an ITT after a 6 h fasting period under fentanyl-midazolam sedation. The reduction of blood glucose levels following the *i.p.* insulin administration within 15 min was significantly greater in the *P4h-tm*^*−/−*^ males and females than in WT, and in females, there were significantly lower fasting glucose levels in the *P4h-tm*^*−/−*^ mice (*P* = 0.004, Fig. 4a

 and *P*  = 0.048, Fig. S4a). As a result, 20–30 min after insulin administration, 60% of the *P4h-tm*^*−/−*^ males and all females lost consciousness which was not observed in the WT mice. Together, these results suggest that both male and female *P4h-tm*^*−/−*^ mice may be more insulin-sensitive than the WT mice. However, these findings may also imply putative alterations in the function of the pituitary and/or adrenal glands, which are also assessed by ITT but were not examined in this study.

### Fat absorption and BAT activity are not altered in *P4h-tm*^*−/−*^ mice

In order to evaluate whether the earlier detected lower serum TG levels in *P4h-tm*^*−/−*^ mice [21] would have been caused by a difference in fat absorption, we carried out an oral fat tolerance test (OFTT) (Supplementary experimental procedures). OFTT showed no differences between the genotypes in the magnitude of the postprandial TG response (Fig. S5a), suggesting that there are no measurable alterations in the intestinal lipid absorption, lipid transport or tissue-specific lipid metabolism in the *P4h-tm*^*−/−*^ mice.

**Fig. 5:**
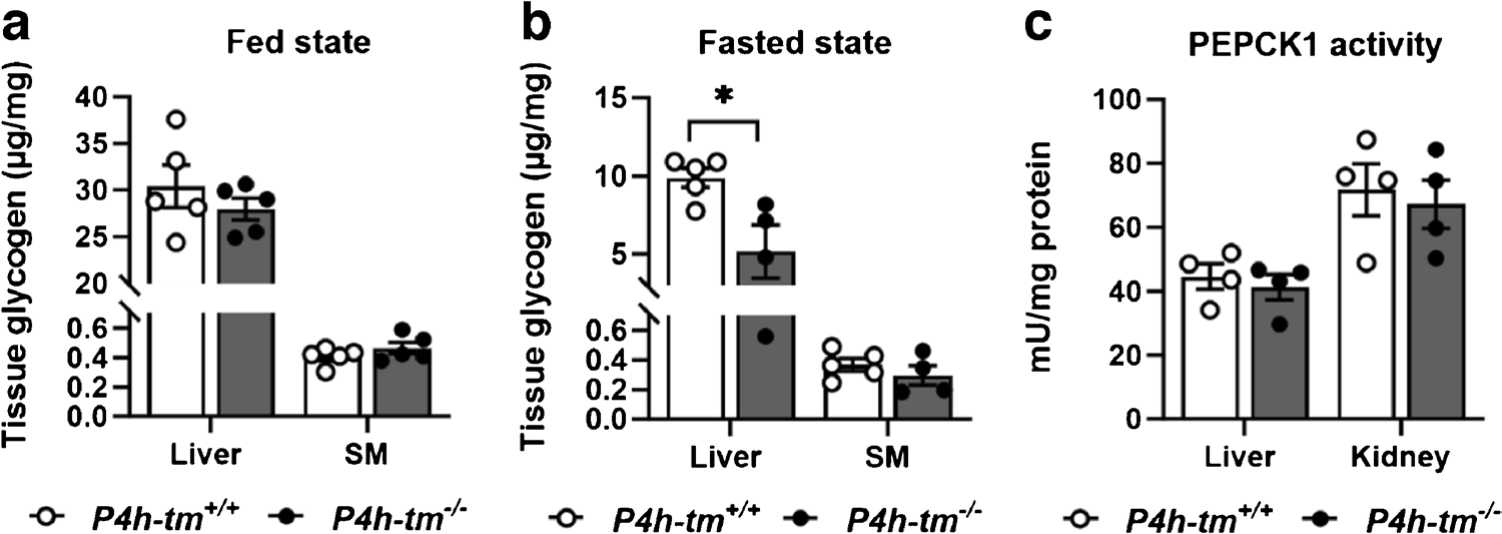
*P4h-tm*^*−/−*^ mice have faster glycogenolysis. Liver and skeletal muscle glycogen levels in 12-month-old male *P4h-tm*^+*/*+^and *P4h-tm*^*−/−*^ mice in (**a**) fed and (**b**) fasted state. (**c**) PEPCK activity in liver and kidney of 2-month-old *P4h-tm*^+*/*+^ and *P4h-tm*^*−/−*^ mice. Data are mean ± SEM. *p ≤ 0.05. *n* = 4–5 *P4h-tm*^+*/*+^*,* *n* = 4–5 *P4h-tm*^*−/−*^

Next, we studied the non-shivering thermogenesis in BAT following norepinephrine (NE) injection in pentobarbital-anesthetized mice (Supplementary experimental procedures). NE led to an increase in body temperature and interscapular BAT (iBAT) temperature over time in both genotypes (Fig. S5b) and to a higher body temperature in the *P4h-tm*^*−/−*^ mice 28 min after injection (Fig. S5b). There was no difference in maximum iBAT temperature (Fig. S5c) or effective thermogenesis (Fig. S5d) between the genotypes, indicating that P4H-TM deficiency has no effect on NE-induced BAT thermogenesis. Serum FFA levels were similar between the genotypes at baseline but lower in *P4h-tm*^*−/−*^ mice after NE administration (*P* = 0.053, Fig. S5e). The baseline FFA levels were inconsistent with our previous findings [21] (Fig. 3d) and may have been influenced by the use of a different anesthetic. Serum free glycerol levels were similar between the genotypes at baseline and increased significantly in both genotypes after NE (*P* = 0.0028 for WT and *P* = 0.0008 for *P4h-tm*^*−/−*^, Fig. 5Sf) indicating a comparable lipolysis. Thus, the differences in serum FFA levels after NE administration may have been caused by increased FFA uptake in *P4h-tm*^*−/−*^ mice compared to WT.

### Hepatic glycogen stores are depleted faster in *P4h-tm*^*−/−*^ mice

Sufficient glycogen stores in the liver are a prerequisite for restoring blood glucose levels in hypoglycemia. Therefore, we analyzed the glycogen levels in liver and skeletal muscle of the WT and *P4h-tm*^*−/−*^ mice in fed and fasted states. There was a difference in the amount of glycogen present in the liver between males and females, males having higher levels (Fig. 5). Additionally, there was a major difference in glycogen levels between the liver and the skeletal muscle (Fig. 5a and Fig. S6a). In the fed state, there was no difference in the amount of glycogen present in liver and skeletal muscle between the genotypes (Fig. 5a and Fig. S6a). After a 6 h fasting period, the amount of liver glycogen decreased to 33% in WT males and 18% in *P4h-tm*^*−/−*^ males (*P* = 0.024, Fig. 5b) whereas no changes in skeletal muscle glycogen levels were observed in either genotype (Fig. 5a,b). At the same time, there was no difference in PEPCK catalytic activity between the genotypes (Fig. 5c). In female mice, 6 h fasting reduced liver glycogen levels to 86% in WT and 43% in *P4h-tm*^*−/−*^ mice (*P* = 0.015, Fig. S6a,b). These data suggest that both male and female *P4h-tm*^*−/−*^ mice have faster hepatic glycogenolysis during fasting.

**Fig. 6:**
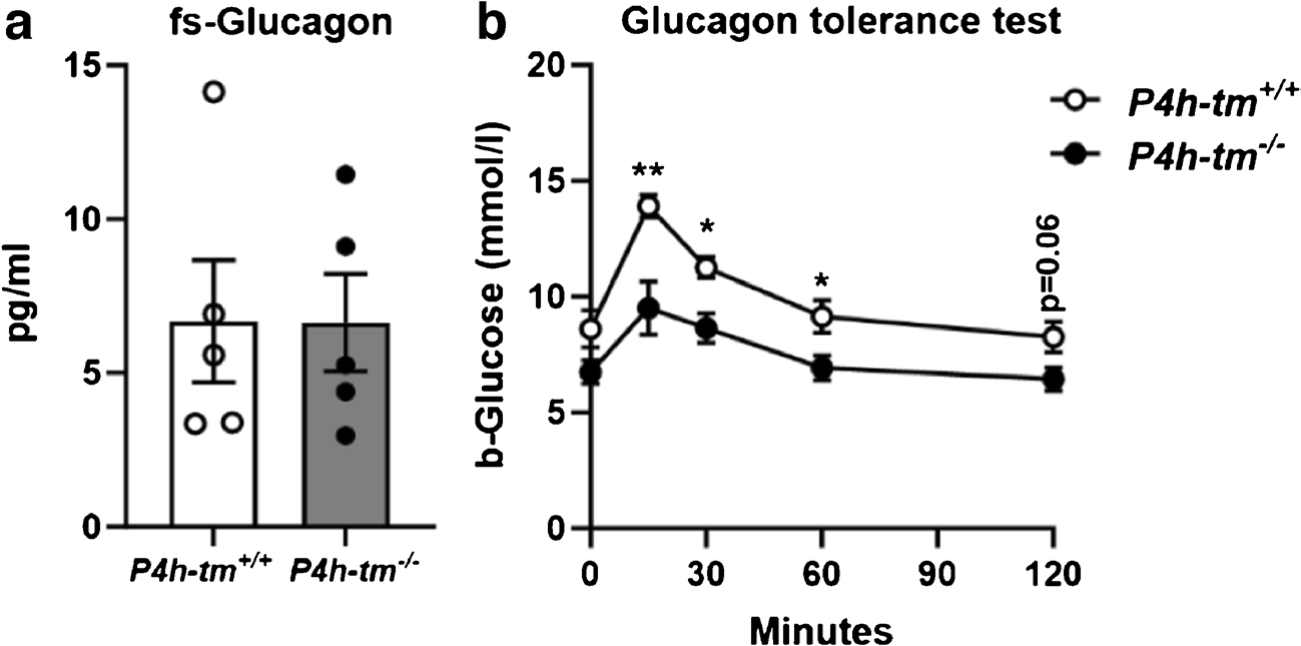
*P4h-tm*^*−/−*^ mice release less glucose in response to exogenous glucagon. (**a**) Fasting serum glucagon levels of 3-month-old *P4h-tm*^+*/*+^and *P4h-tm*^*−/−*^ male mice. (**b**) Glucagon tolerance test. The 0 min value was determined after 6 h fasting. Data are mean ± SEM. *p ≤ 0.05, **p < 0.01. *n* = 5 *P4h-tm*^+*/*+^, *n* = 5 *P4h-tm*^*−/−*^

To investigate further responses on glucose levels, we assessed the whole-body glucagon response in non-anesthetized mice after a 6 h fasting period and an intraperitoneal glucagon injection. No differences in fasting glucagon levels were detected between the genotypes at baseline in either sex (Fig. 6a, Fig. S7a). Exogenously administered glucagon increased the blood glucose levels in both male and female WT and *P4h-tm*^*−/−*^ mice; this increase was significantly lower by ~ 25% in *P4h-tm*^*−/−*^ males than in WT at all measured time points (*P* = 0.0081 for 15 min, *P* = 0.011 for 30 min, *P* = 0.036 for 60 min, and *P* = 0.06 for 120 min, Fig. 6b). In female mice, the difference in blood glucose between genotypes was significant only after 15 min of glucagon administration (*P* = 0.012, Fig.S7b). These results are consistent with the observed lower hepatic glycogen levels of *P4h-tm*^*−/−*^ mice after 6 h fasting.

### Alterations in glucose tolerance are not present in non-anesthetized *P4h-tm*^*−/−*^ mice

**Fig. 7:**
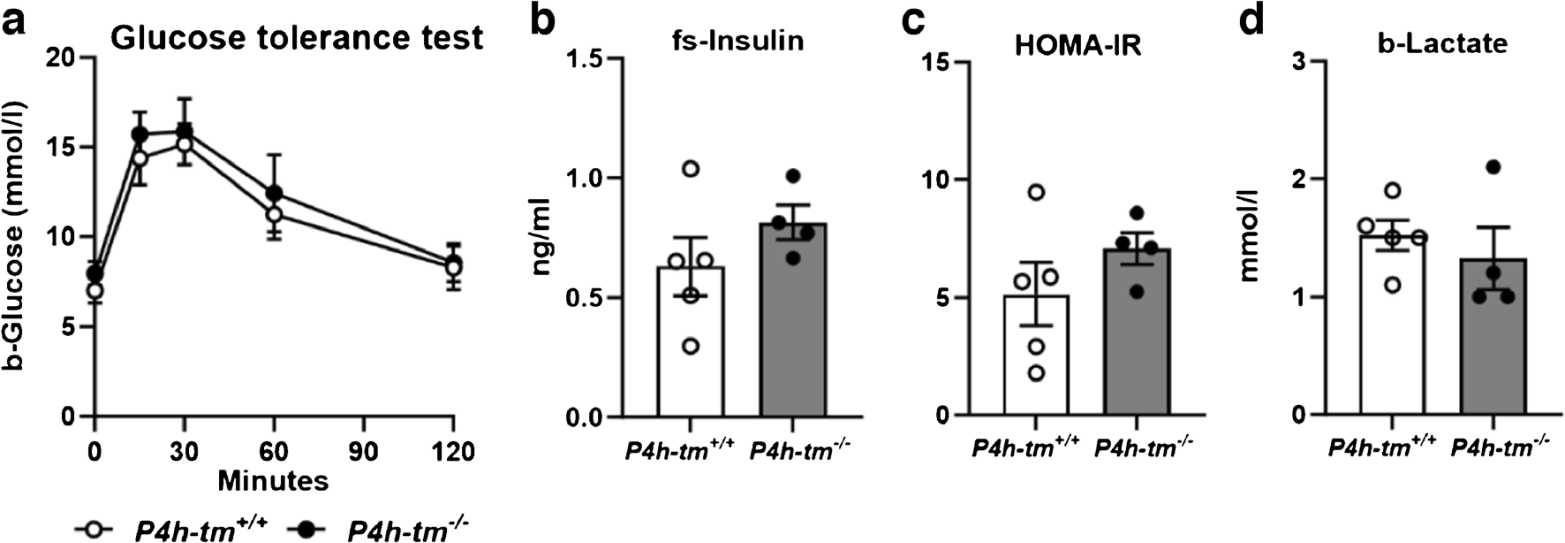
Conscious *P4h-tm*^*−/−*^ do not show improved glucose tolerance, better insulin sensitivity or increased blood lactate levels. (**a**) Glucose tolerance test of 5-month-old *P4h-tm*^+*/*+^and *P4h-tm*^*−/−*^ male mice. The 0 min value was determined after 12 h fasting. (**b**) Fasting serum insulin levels, and (**c**) HOMA-IR scores were determined from the 0 min samples. (**d**) Fasting blood lactate levels. Data are mean ± SEM. *n* = 5 *P4h-tm*^+*/*+^*,* *n* = 4 *P4h-tm*^*−/−*^*.* b, blood, fs, fasting serum

When non-anesthetized 5-month-old male and 7-month-old female mice were subjected to an intraperitoneal GTT after an overnight fast, surprisingly, there were no differences in fasting glucose levels, glucose tolerance, fasting insulin levels, HOMA-IR scores or fasting blood lactate levels between the genotypes at either gender (Fig. 7a-d and Fig. S8a-d). Thus, the data suggest that the difference in glucose tolerance is substantially impacted by fentanyl-midazolam sedation, particularly in *P4h-tm*^*−/−*^ mice, which exhibited a heightened susceptibility to it.

### Reduced respiratory rate and acidosis in *P4h-tm*^*−/−*^ mice

**Fig. 8:**
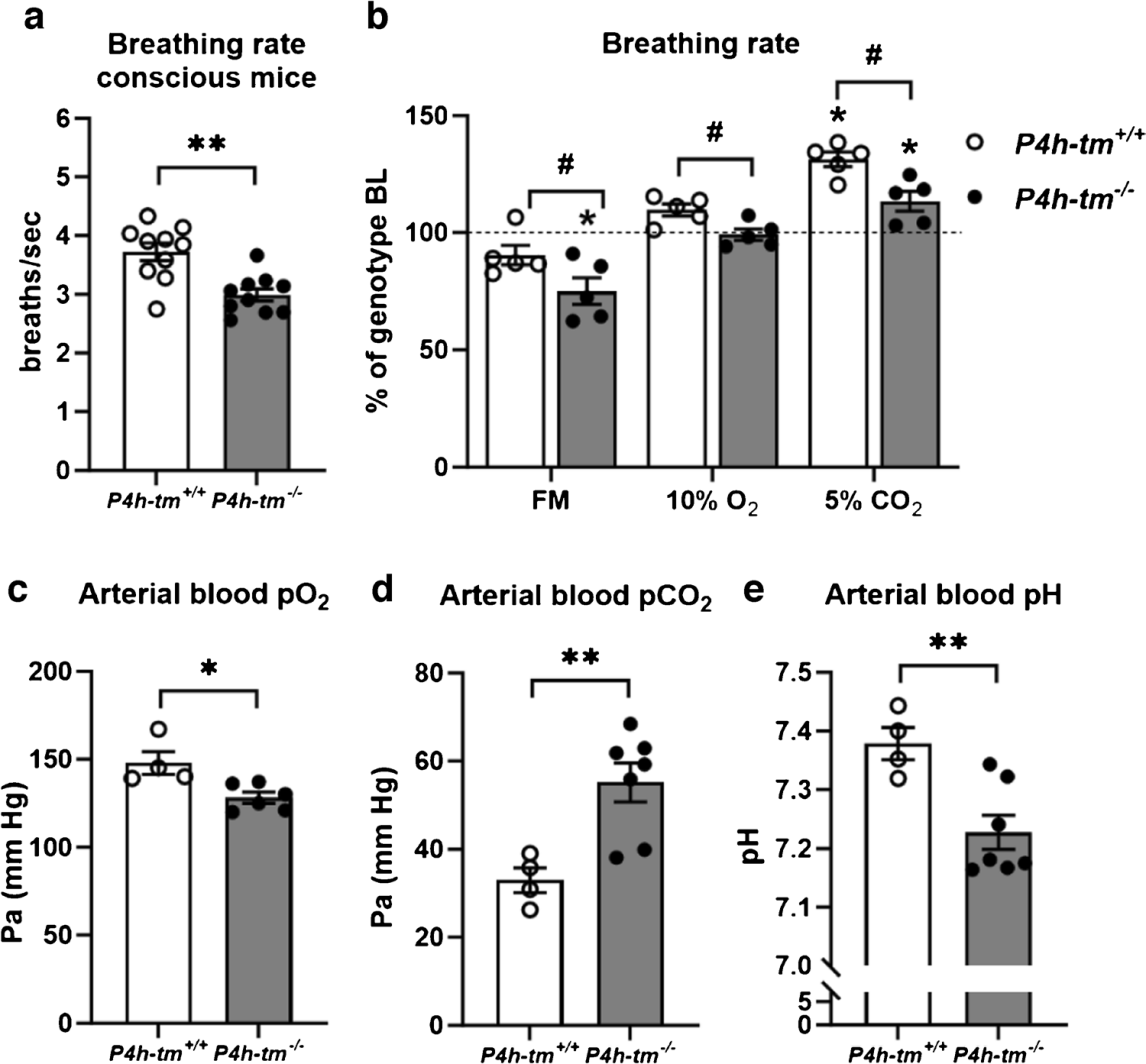
*P4h-tm*^*−/−*^ mice have a reduced respiratory rate, reduced response to hypoxia and hypercapnia and respiratory acidosis. The respiratory rate in (**a**) conscious mice (**b**) mice sedated with fentanyl-midazolam mice and conscious mice under hypoxia (10% O_2_) and hypercapnia (5% CO_2_). (**c-e**) Arterial blood was drawn through a catheter in the femoral artery. (**c**) Arterial blood oxygen partial pressure. (**d**) Arterial blood carbon dioxide pressure. (**e**) Arterial blood pH. Data are mean ± SEM. *p ≤ 0.05, **p < 0.01, # significant difference between *P4h-tm*^+*/*+^ and *P4h-tm*^*−/−*^. FM, fentanyl-midazolam; *n* = 4–10 *P4h-tm*^+*/*+^*,* *n* = 5–10 *P4h-tm*^*−/−*^

Given the diverged metabolic responses in conscious *vs.* sedated *P4h-tm*^*−/−*^ mice and considering that the HIDEA patients are characterized by hypoventilation and sleep apnea [15], we investigated the breathing of the mice. The respiratory rate of conscious *P4h-tm*^*−/−*^ male mice was significantly 20% lower compared to the WT mice (*P* = 0.008, Fig. 8a). When respiratory rate was analyzed under fentanyl-midazolam sedation, the respiratory rate of *P4h-tm*^*−/−*^ males was further reduced by 25% compared to conscious *P4h-tm*^*−/−*^ mice (*P* = 0.018), whereas this reduction was only 10% in WT mice (*P* = 0.98) (Fig. 8b). The reduced respiratory rate under sedation was also associated with lower arterial blood pO_2_ (*P* = 0.016), higher arterial blood pCO_2_ (*P* = 0.007) and lower arterial pH (*P* = 0.007) in the *P4h-tm*^*−/−*^ males compared to WT (Fig. 8c-e). We also assessed the response of the respiratory rate in conscious mice to environmental hypoxia and hypercapnia. Hypoxia (10% oxygen) increased the respiratory rate of the WT mice by 11% (although this did not reach statistical significance), whereas the respiratory rate of the *P4h-tm*^*−/−*^ mice was unchanged (Fig. 8b). Hypercapnia (5% CO_2_) significantly increased the respiratory rate of the WT mice by 31% (*P* = 0.039) whereas the *P4h-tm*^*−/−*^ mice showed only a 13% increase (*P* = 0.036) (Fig. 8b). Thus, the *P4h-tm*^*−/−*^ mice have a decreased respiratory rate which is exacerbated by sedation/anesthesia and is associated with acidosis and a reduced ventilatory response to both hypoxia and hypercapnia.

## Discussion

We report here the metabolic characterization of *P4h-tm*^*−/−*^ mice. We found that the global deficiency of P4H-TM in mice is associated with alterations in their whole-body energy, day-night rhythm of activity, glucose homeostasis, neuromuscular and respiratory functions. Although the exact underlying mechanism(s) remain to be identified, they appear to be neurological and controlled by the brain and central nervous system circuits. Importantly, P4H-TM deficiency in mice recapitulates some of the symptoms seen in HIDEA patients, making this mouse model a valuable tool to study, advance knowledge and develop tailored therapies for the disease.

Our previous work showed that P4H-TM deficiency coincides with lower serum TG levels on both a standard chow and a high-fat diet [21], suggesting an altered energy metabolism. Intriguingly, *P4h-tm*^*−/−*^ mice showed only subtle variations in the 24-h oscillation of energy expenditure, O_2_ consumption and CO_2_ production in home cage analysis that manifested with increased frequency and reduced amplitude. As the rhythm of the last two parameters is controlled by the circadian clock [1] and all these parameters themselves are indicators of metabolic rate and fuel utilization, it is tempting to speculate that P4H-TM deficiency associates with a subtle disruption of circadian rhythm and/or energy metabolism. Furthermore, the locomotor activity of *P4h-tm*^*−/−*^ mice was reduced during the night, the normally active time for mice, and increased during the day, suggesting that their day-night rhythm of activity is disrupted, which may also indicate altered circadian rhythm. There is currently no evidence linking circadian rhythm to P4H-TM or HIDEA patients. However, an association between circadian rhythm, neurodevelopmental disorders and neurogenerative diseases has been suggested [19]. Interestingly, patients with neurodegenerative diseases often have impaired sleep and alertness, and circadian rhythm disturbances manifest as increased activity at night and less activity during the day [30, 31]. While such circadian dysfunction is common in older adults and is partly attributed to the degeneration of the suprachiasmatic nucleus (SCN), known as the master circadian clock, the link between circadian rhythms and neurodegeneration is not well understood [19]. It is important to note that P4H-TM is highly expressed in the entire hypothalamus [17], where the SCN are localized [5, 6, 25]. However, the existence and causal relationship between P4H-TM and circadian rhythm remains to be established. Identification of the primary P4H-TM substrate is expected to provide a better understanding of this phenotype. The only substrate identified so far, HIF1α [14, 17, 18] is the primary substrate for HIF-P4Hs 1–3. Stabilization of different HIFαs due to HIF-P4H-1–3 deficiency contribute to glucose metabolism ([29] and references therein) but the effects are differential to those seen in *P4h-tm*^*−/−*^ mice here.

The previously observed locomotor hyperactivity of *P4h-tm*^*−/−*^ mice [18], and the reduced ability to stand on their hind legs or run on a treadmill observed in this study, may be indicative of muscle and/or motor dysfunction. Therefore, we investigated the motor function, coordination, balance, and sensory deficits in *P4h-tm*^*−/−*^ mice using a grid-hanging test, rotarod, catwalk analyses and treadmill running. *P4h-tm*^*−/−*^ mice exhibited significant muscle weakness and motor dysfunction in all these tests. Our findings suggest that P4H-TM plays a role in normal motor function in mice and that its deficiency leads to hypotonia as seen in HIDEA patients [9, 15, 24]. Although correlational data suggest a potential link between circadian rhythm irregularities and motor dysfunction, particularly in the context of neurological and brain disorders [19, 20], the causal relationship, both generally and specifically, between the muscle phenotype of the *P4h-tm*^*−/−*^ mice and their disrupted day-night activity rhythm, is as yet unclear.

In sedated *P4h-tm*^*−/−*^ mice, glucose metabolism indices showed significant changes: fasting insulin levels were lower, glucose and insulin tolerance tests showed improved outcomes. However, over 50% of the *P4h-tm*^*−/−*^ mice lost consciousness ~ 30 min after an intraperitoneal insulin injection. Although the cause remains unknown, it may be due to a seizure, as seen in some HIDEA patients [9, 15, 23] or/and hypoglycemic shock, at least partly explained by their lower glycogen content in the liver after fasting. In line with this, the blood glucose levels after the mobilization of glycogen from the liver in response to exogenous glucagon were lower in *P4h-tm*^*−/−*^ mice than wild type. In addition, the sedated *P4h-tm*^*−/−*^ mice had elevated fasting blood lactate levels. Given the observed hypoventilation, an obvious explanation appears to be tissue hypoxia and hence increased glycolytic metabolism. However, we cannot exclude the possibility that other processes unrelated to tissue oxygenation such as acute energy demand or alterations in lactate clearance [3] may have led or at least partly contributed to the increased lactate levels in the *P4h-tm*^*−/−*^ mice under sedation.

Notably, conscious *P4h-tm*^*−/−*^ mice did not show improved glucose tolerance or better insulin sensitivity, nor increased blood lactate levels, suggesting that the metabolism of *P4h-tm*^*−/−*^ mice is majorly altered by fentanyl-midazolam sedation. Sedated *P4h-tm*^*−/−*^ mice appeared to have a higher substrate requirement than conscious *P4h-tm*^*−/−*^ mice leading to a faster clearance rate of exogenous/endogenous glucose and FFA from the circulation, suggesting a decrease in effective tissue oxidative energy metabolism under sedation.

In this study we also identified phenotypic changes related to the respiration in P*4h-tm*^*−/−*^ mice which are closely related to breathing disorders such as hypoventilation and obstructive sleep apnea, commonly observed in HIDEA patients [9, 15, 24]. Conscious *P4h-tm*^*−/−*^ mice showed subtle fluctuations in 24-h oscillation of oxygen and carbon dioxide during the indirect calorimetry, as well as a significant reduction in respiratory rate. However, an additional decrease in respiratory rate observed in sedated *P4h-tm*^*−/−*^ mice that could not be compensated for, resulted in respiratory acidosis, rapid energy substrate utilization and a decrease in insulin levels. In addition, *P4h-tm*^*−/−*^ mice had reduced breathing responses to hypoxia and hypercapnia further linking the deficiency of P4H-TM to respiratory activity.

The respiratory center, located in the *medulla oblongata* and pons of the brainstem, is responsible for generating, maintaining, and sustaining the respiratory rhythm while adapting to physiological changes. It receives an input from chemo- and mechanoreceptors, the cerebral cortex (voluntary breathing), and the hypothalamus (emotional and hormonal responses) to regulate the breathing rate and depth [7]. However, the molecular level relationship between P4H-TM deficiency and breathing remains unclear. Future research needs to answer the question of whether P4H-TM deficiency directly affects the function of the brainstem or central or peripheral respiratory chemoreceptors, or whether it affects the transmission of signals from the chemoreceptors to the CNS.

Our study found sex differences in some parameters and tests, but not in others. However, since HIDEA patients do not exhibit any sex-specific phenotype, the significance of these differences seems to be of minor importance. As the mouse model recapitulates some of the symptoms of the HIDEA patients, the focus of this study remains on the effects of P4H-TM deficiency.

All in all, *P4h-tm*^*−/−*^ mice display alterations in energy metabolism, day-night rhythm of activity, glucose metabolism, neuromuscular function and respiration. This phenotype most likely originates from disturbances in the brain and/or central nervous system circuits. The data reported herein provide additional insights into the role of P4H-TM in metabolism and physiology.

### Supplementary Information

Below is the link to the electronic supplementary material.

Supplementary file1 (DOCX 2281 KB)

Supplementary file2 (MOV 1158 KB)

Supplementary file3 (MOV 1525 KB)

## Data Availability

No datasets were generated or analysed during the current study.
