# Supplementary material for: Metabolic characteristics of transmembrane prolyl 4-hydroxylase (P4H-TM) deficient mice
Source: Pflugers Arch. 2024 Feb 24;476(9):1339–51. doi: 10.1007/s00424-024-02920-5 (PMC11310233; doi:10.1007/s00424-024-02920-5)
Supplement: Supplementary file 1 — Supplementary file1 (DOCX 2281 KB) [file 424_2024_2920_MOESM1_ESM.docx]

**Supplementary material**

**Metabolic characteristics of transmembrane prolyl 4-hydroxylase (P4HTM) deficient mouse model**

Tuulia Ala-Nisula^1^, Riikka Halmetoja^1^, Henri Leinonen^2,3^, Margareta Kurkela^1^, Henna-Riikka Lipponen^2^, Samuli Sakko^1^, Mikko Karpale^1^, Niina Sissala^1^, Tapio Röning^1^, Ghulam S. Raza^3^, Kari A. Mäkelä^4^, JérômeThevenot^5^, Karl-Heinz Herzig^4^, Raisa Serpi^1^, Johanna Myllyharju^1^, Heikki Tanila^2^, Peppi Koivunen^1*^ and Elitsa Y. Dimova^1*^

*Equal senior authors

^1^Biocenter Oulu, Faculty of Biochemistry and Molecular Medicine, Oulu Center for Cell-Matrix Research, University of Oulu, Oulu, Finland. ^2^A.I. Virtanen Institute for Molecular Sciences, University of Eastern Finland, Kuopio, Finland. ^3^School of Pharmacy, University of Eastern Finland, Kuopio, Finland. ^3^Research Unit of Biomedicine and Internal Medicine, Biocenter Oulu, Medical Research Center and University Hospital, Oulu, Finland. ^5^Research Unit of Health Sciences and Technology, University of Oulu, Oulu, Finland

Short title: P4H-TM and metabolism

Correspondence to: Peppi Koivunen, MD, PhD, Faculty of Biochemistry and Molecular Medicine, University of Oulu, Aapistie 7C, P.O. Box 5400, FIN-90014, Finland, Email [peppi.koivunen@oulu.fi](mailto:peppi.koivunen@oulu.fi),Tel. +358-8-5375822

**Supplementary experimental procedures**

**Oral fat tolerance test**

Oral fat tolerance test (OFTT) was performed on 4-5-month-old male mice after a 4 h fast without sedation. For the OFTT, mice were given 200 µl olive oil (~ 7 ml/kg) via orogastric gavage and blood samples were taken from the mouse tail by the tail-snip method at 30, 60, 90, 180 and 240 min time points. Serum TG levels were determined by an enzymatic method (Roche Diagnostics) and the absorbance of the colorimetric products was determined with the Infinite M1000 Pro Multimode Plate Reader (Tecan).

**Thermoimaging**

Thermal imaging experiment was performed on male mice at the age of 8 months after a 12 h overnight fast. To minimize stress and to keep the distance between the mice and the thermal camera constant between each data collection, mice were anesthetized with pentobarbital (Mebunat, IP 60mg/kg) [1], one of the few anesthetics known not to impair BAT function [2]. The mice backs were shaved beforehand from between the ears to the lumbar area. Baseline blood samples were taken from *vena saphena* of the hind leg and blood glucose was measured. The mice were injected with norepinephrine (1mg/kg) subcutaneously and thermal images were taken from the mouse BAT up to 35 minutes once every minute with a thermal camera (Flir T420 thermal camera, FLIR Systems, Oregon, USA). The mice were sacrificed after imaging and tissues were collected.

**Determination of free glycerol levels**

The serum free glycerol levels were determined following manufacturer’s instructions with Free Glycerol Assay Kit (ab65337, Abcam).

**Supplementary references**

1. Abreu-Vieira G, Hagberg CE, Spalding KL, Cannon B, Nedergaard J (2015) Adrenergically stimulated blood flow in brown adipose tissue is not dependent on thermogenesis. Am J Physiol Endocrinol Metab. 308:E822-9. <https://doi.org/10.1152/ajpendo.00494.2014>

2. Ohlson KB, Lindahl SG, Cannon B, Nedergaard J (2003) Thermogenesis inhibition in brown adipocytes is a specific property of volatile anesthetics. Anesthesiology. 98:437-48. <https://doi.org/10.1097/00000542-200302000-00025>

**Supplementary figure legends**

**Fig. S1 Body and organ weights of *P4h-tm^+/+^*and *P4h-tm^-/-^* male and female mice** Body weight development of *P4h-tm^+/+^* and *P4h-tm^-/-^* (**a**) male and (**b**) female mice fed normal chow and the gonadal WAT, liver kidney, spleen and BAT weights of 1-year-old mice. (Males: n=12 *P4h-tm^+/+^*, n=13 *P4h-tm^-/-^*, females: n=7 *P4h-tm^+/+^*, n=8 *P4h-tm^-/-^).* Data are means ± SEM. **p*≤0.05. BAT, brown adipose tissue; WAT, white adipose tissue;

**Fig. S2** ***P4h-tm^-/-^* female mice show subtle variations in frequency and reduced amplitude in circadian oscillations of energy expenditure, locomotor and rearing activity, O_2_ consumption and CO_2_ production and appear to be more active during the day (a-g)** Metabolic profiling of 8-month-old female mice housed in a home cage phenotyping system for 7 days at room temperature and a normal light/dark rhythm. (**a**) Energy expenditure (EE), (**b**) Locomotor activity, (**c**) Rearing activity, (**d**) O_2_ consumption (VO_2_), (**e**) CO_2_ production (VCO_2_), (**f**) Food intake. (**g**) Water intake. Data are means ± SEM. **p*≤0.05, n=4 *P4h-tm^+/+^*, n=4 *P4h-tm^-/-^.*

**Fig. S3 Changes in glucose metabolism in sedated *P4h-tm^-/-^* female mice (a)**: GTT of 12-month-old female *P4h-tm^+/+^*and *P4h-tm^-/-^* mice. The 0 min value was determined after 12 h fasting and sedation with fentanyl-midazolam. (**b**) Serum insulin levels, and (**c**) HOMA-IR values determined from 0 min samples. (**d**) Fasting blood lactate levels. (**e**) Fasting serum FFA levels. Data are means ± SEM. ****p*<0.001. n=8 *P4h-tm^+/+^*, n=8 *P4h-tm^-/-^*.

**Fig. S4 Increased insulin sensitivity in *P4h-tm^-/-^* female mice** Insulin tolerance test of 6-month-old female *P4h-tm^+/+^*and *P4h-tm^-/-^* mice. The 0 min value was determined after fasting for 6 h and sedation with fentanyl-midazolam. Data are means ± SEM. *p*≤0.05. n=5 *P4h-tm^+/+^*, n=5 *P4h-tm^-/-^.*

**Fig. S5 *P4h-tm^-/-^* mice do not show alterations in lipid absorption and norepinephrine (NE)-induced brown adipose tissue (BAT) thermogenesis (a)** Fat tolerance test in 12 h fasted *P4h-tm^+/+^* and *P4h-tm^-/-^* male mice. The mice were oral gavaged with 200 µl olive oil and blood samples were collected at the indicated times. n=8 *P4h-tm^+/+^,* n=9 *P4h-tm^-/-^*. **(b-d)** Functional analysis of BAT thermogenic capacity following a NE injection (1 mg/kg) in 12 h fasted *P4h-tm^+/+^* and *P4h-tm^-/-^* male mice housed at 21°C and anesthetized with pentobarbital. (**b**) Reference surface body temperature, (**c**) Max interscapular BAT (iBAT) temperature, (**d**) Effective thermogenesis calculated by subtracting maximum iBAT from the reference surface body temperature, (**e**) Fasting serum free fatty acid (FFA) levels. (**f**) Fasting serum free glycerol levels. Data mean ± SEM. **p*≤0.05, ***p*<0.01, ****p*<0.001. n=5 *P4h-tm^+/+^,* n=6 *P4h-tm^-/-^.* BL, baseline.

**Fig. S6 *P4h-tm^-/-^* female mice have faster hepatic glycogenolysis during fasting** Liver and skeletal muscle glycogen levels in 12-month-old female *P4h-tm^+/+^*and *P4h-tm^-/-^* mice in (**a**) fed (n=6 *P4h-tm^+/+^*, n=6 *P4h-tm^-/-^.* and (**b**) fasted state n=7 *P4h-tm^+/+^*, n=8 *P4h-tm^-/)^*. Data are means ± SEM. **p*≤0.05.

**Fig. S7 Glucagon levels and glucagon tolerance in *P4h-tm^-/-^* female mice (a)** Fasting serum glucagon levels. (**b**) Glucagon tolerance test of 4-month-old female *P4h-tm^+/+^*and *P4h-tm^-/-^* mice. The 0 min value was determined after fasting for 6 h. Data are means ± SEM. n=6 *P4h-tm^+/+^*, n=6 *P4h-tm^-/-^.*

**Fig. S8** **Conscious *P4h-tm^-/-^* have do not show improved glucose tolerance and insulin sensitivity** (**a**) Glucose tolerance test of 7-month-old female *P4h-tm^+/+^*and *P4h-tm^-/-^* mice. The 0 min value was determined after 12 h fasting. (**b**) Fasting serum insulin levels, and (**c**) HOMA-IR values determined from the 0 min samples. Data are means ± SEM. n=6 *P4h-tm^+/+^*, n=6 *P4h-tm^-/-^.* b, blood, fs, fasting serum.

**Supplementary figures**

| **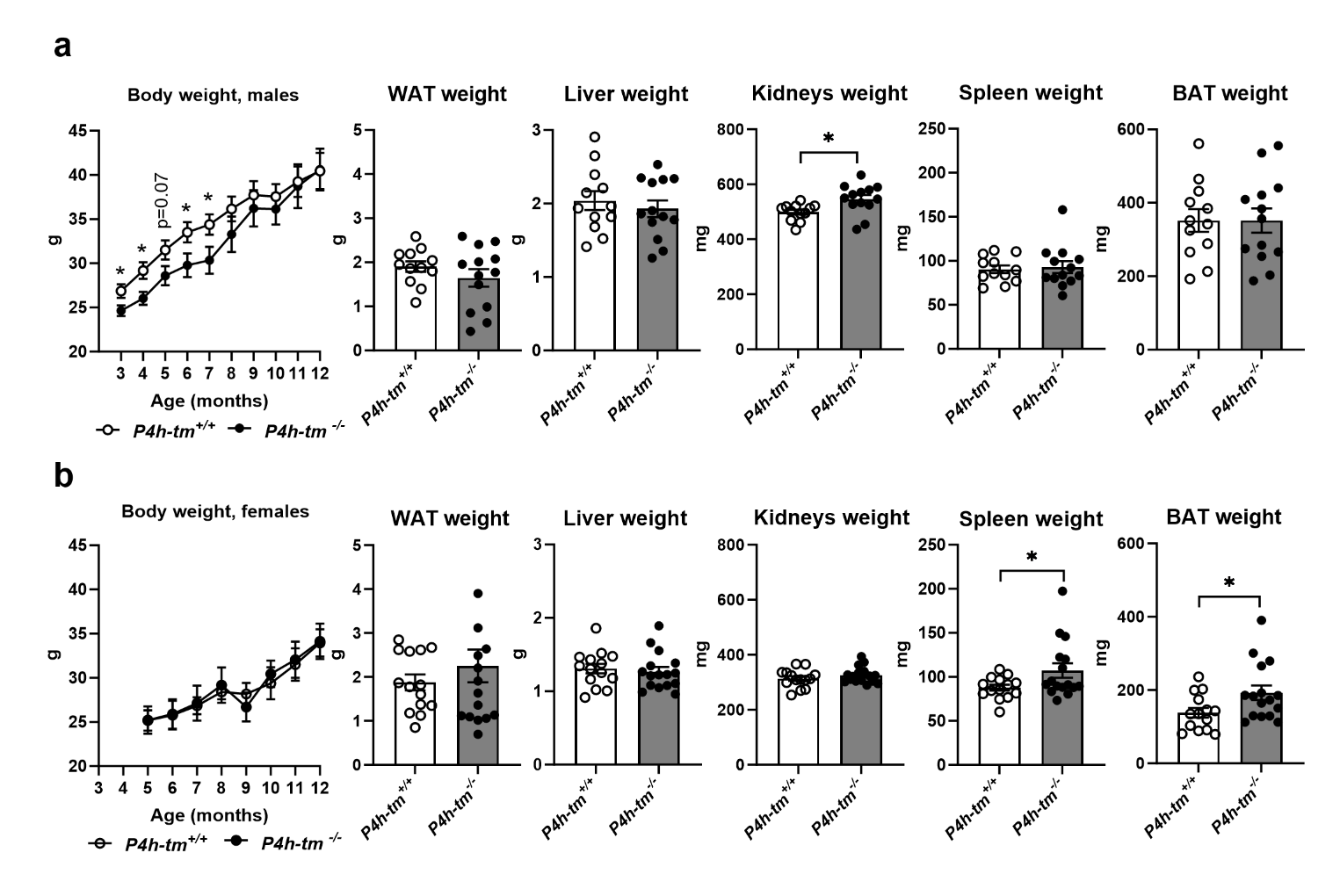** |
| --- |
| Fig. S1 |
| 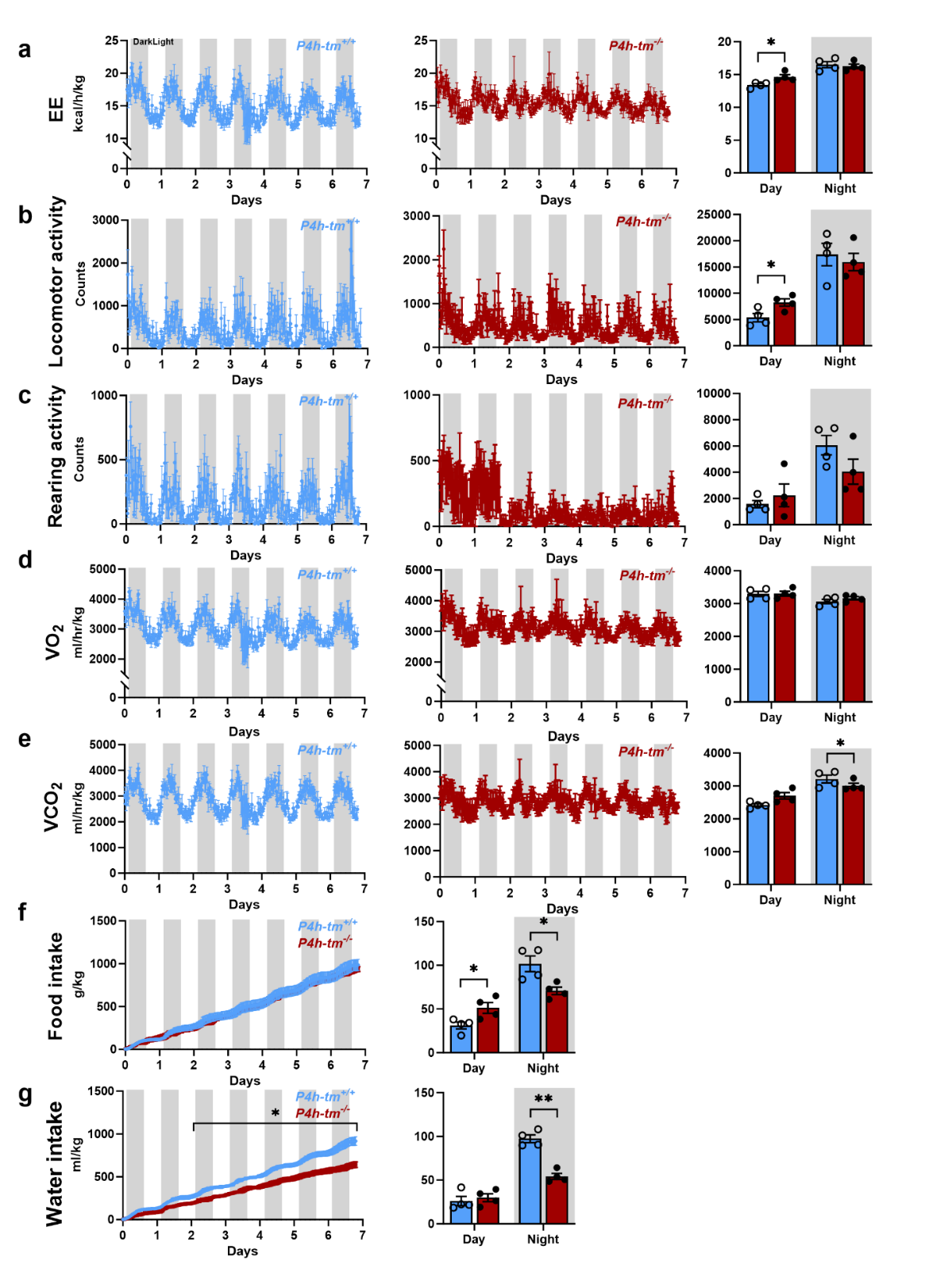 |
| Fig. S2 |
| 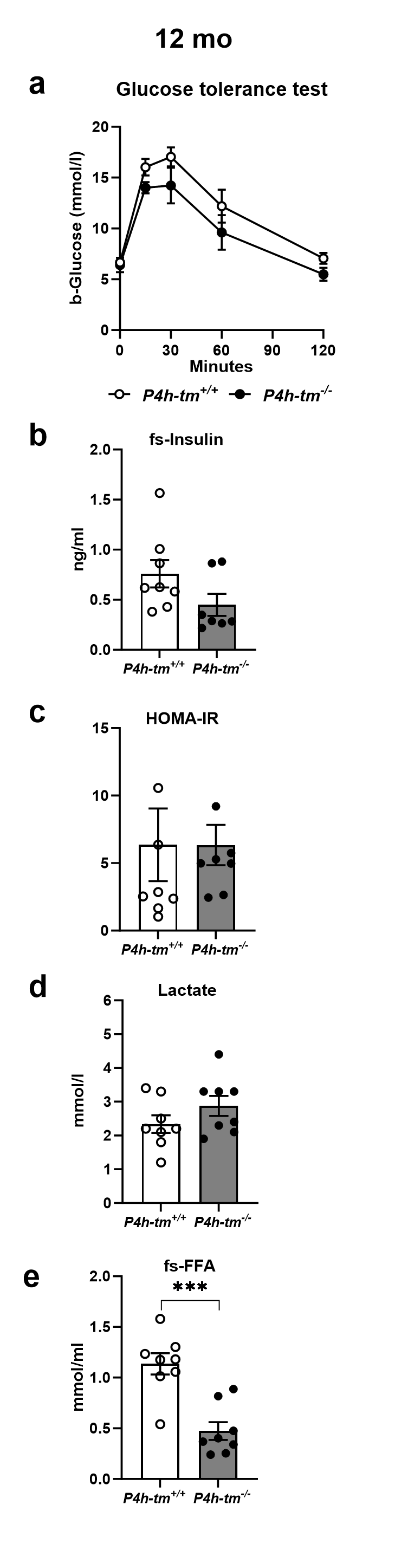 |
| Fig. S3 |
| 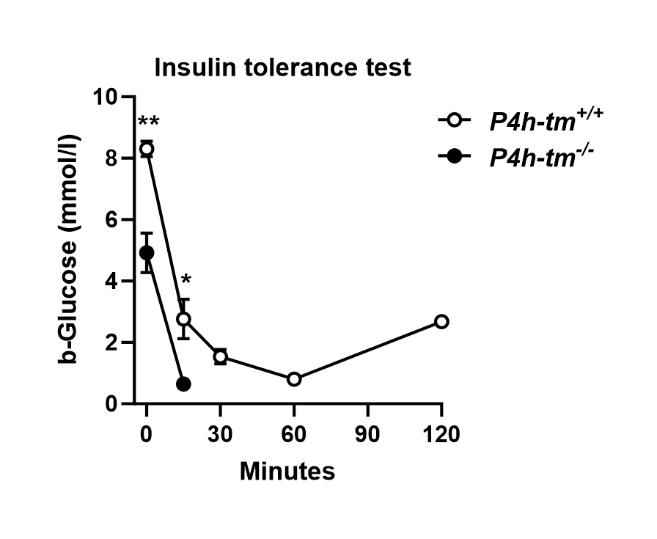 |
| Fig. S4 |

| 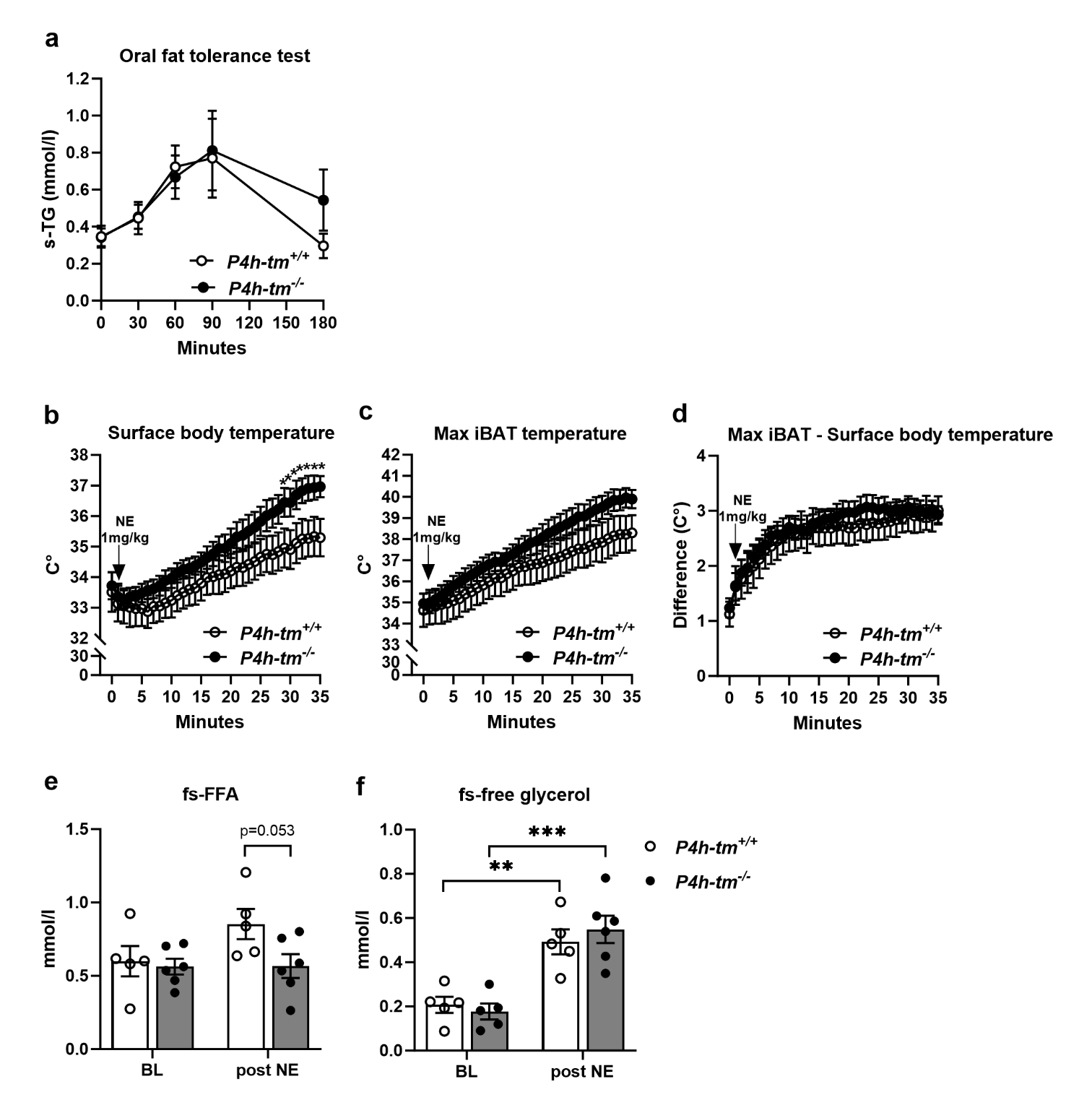 |
| --- |
| Fig. S5 |

| 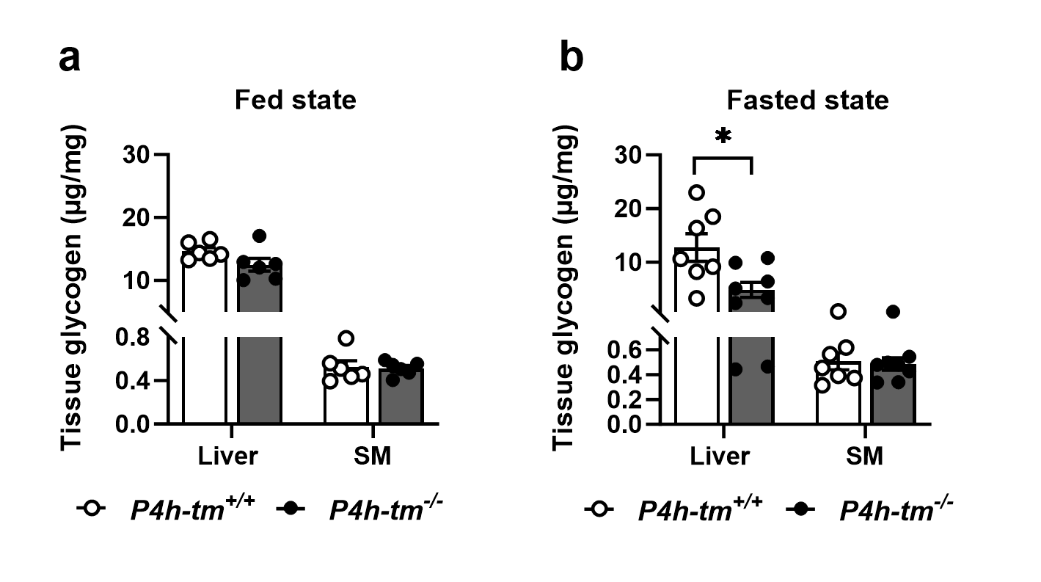 |
| --- |
| Fig. S6 |

| 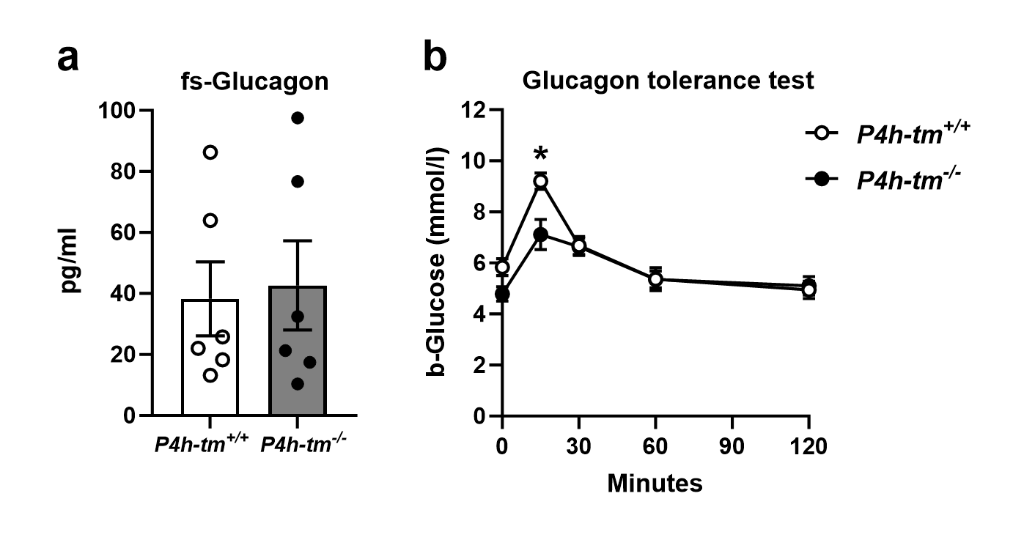 |
| --- |
| Fig. S7 |

| 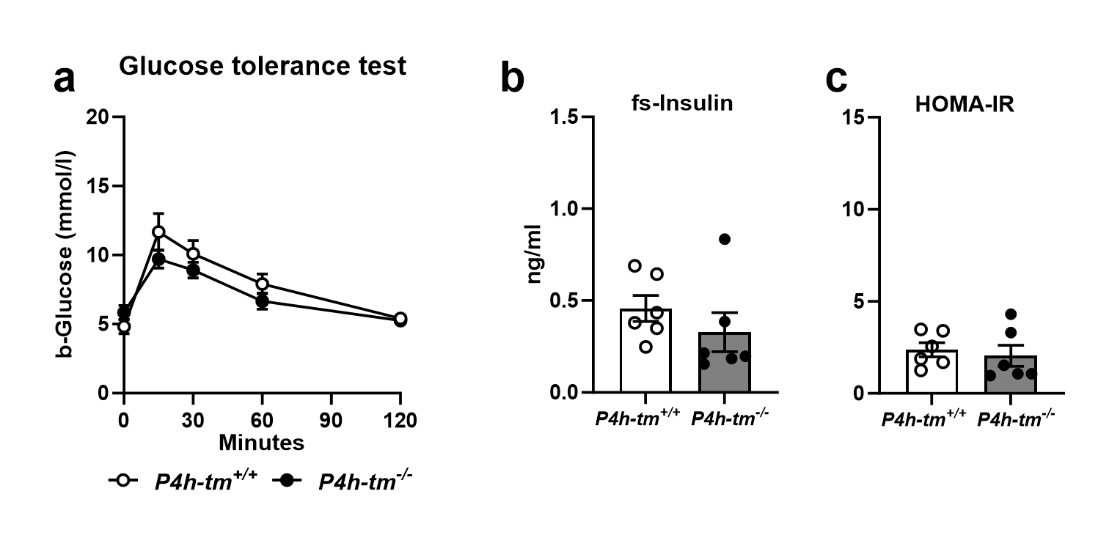 |
| --- |
| Fig. S8 |
